# The value of repeat kidney biopsy during an atypical course of membranous nephropathy

**DOI:** 10.1186/s12882-022-02863-y

**Published:** 2022-07-07

**Authors:** Sumaiya Ahmed, David Massicotte-Azarniouch, Mark Canney, Clare Booth, Paula Blanco, Gregory L. Hundemer

**Affiliations:** 1 Department of Medicine, Ottawa Hospital, University of Ottawa, Ottawa, ON Canada; 2 Department of Medicine, Division of Nephrology, University of Ottawa, Ottawa, ON Canada; 3 Clinical Epidemiology Program, Ottawa Hospital Research Institute, The Ottawa Hospital, University of Ottawa, Riverside Campus, 1967 Riverside Drive, Ottawa, ON K1H 7W9 Canada; 4 Deparment of Pathology and Laboratory Medicine, Ottawa Hospital, University of Ottawa, Ottawa, ON Canada

**Keywords:** Membranous nephropathy, Nephrotic syndrome, Post-infectious glomerulonephritis, Kidney biopsy, Case report

## Abstract

**Background:**

The clinical trajectory for patients with primary membranous nephropathy ranges widely from spontaneous remission to a rapid decline in kidney function. Etiologies for rapid progression with membranous nephropathy include concurrent bilateral renal vein thrombosis, malignant hypertension, and crescentic membranous nephropathy. Given the wide heterogeneity in prognosis, timing of immunosuppressive therapy is often challenging and centers around an individual patient’s perceived risk for rapidly progressive disease.

**Case presentation:**

Herein, we describe the clinical course of a young patient who initially developed a typical presentation of membranous nephropathy with consistent kidney biopsy findings. Given clinical stability, a six month observation period was undertaken prior to initiating immunosuppression. Within this observation window, the patient developed community acquired pneumonia followed several weeks later by a sudden, rapid decline in kidney function requiring dialysis. Repeat kidney biopsy revealed post-infectious glomerulonephritis superimposed upon a background of membranous nephropathy. Immunosuppressive therapy resulted in a favorable long-term outcome with normalization of kidney function and remission of nephrotic syndrome. To our knowledge, this is the first report of the simultaneous occurrence of these two glomerular disease processes.

**Conclusion:**

This case illustrates the value of repeat kidney biopsy during an atypical course of membranous nephropathy. Superimposed glomerular disease processes should be considered during a course of rapidly progressive membranous nephropathy.

**Supplementary Information:**

The online version contains supplementary material available at 10.1186/s12882-022-02863-y.

## Background

Membranous nephropathy is among the most common causes of nephrotic syndrome in adults. The majority of cases are primary and related to auto-antibodies against podocyte antigens, most notably phospholipase A2 receptor (PLA2R) and thrombospondin type-1 domain-containing 7A (THSD7A) though newer antigens continue to be discovered [1]. Secondary causes include systemic diseases such as systemic lupus erythematosus and IgG4-related disease, solid tumor malignancies, chronic infections, and drugs. The clinical course for membranous nephropathy ranges widely from spontaneous remission to accelerated decline in kidney function. Etiologies for accelerated decline in kidney function include concurrent bilateral renal vein thrombosis [2], malignant hypertension [3], and crescentic membranous nephropathy which is often associated with antineutrophil cytoplasmic antibodies (ANCA), anti-glomerular basement membrane (GBM) antibodies, and other auto-antibodies [4–6]. Given the heterogeneous severity in clinical course for patients with primary membranous nephropathy, the decision on whether to start immunosuppressive therapy upon presentation or allow for an initial observation period is often challenging and centers around the perceived risk for accelerated disease [7].

In this report, we describe a case of biopsy-proven membranous nephropathy where an initial observation period was undertaken during which the patient developed an accelerated decline in kidney function. We discuss the value of repeat kidney biopsy in identifying the etiology of the accelerated decline and explore the pros and cons of early immunosuppressive therapy in membranous nephropathy.

## Case presentation

A 38-year-old woman with a history of Crohn’s disease and a recent diagnosis of biopsy-proven membranous nephropathy with left renal vein thrombosis, presented with severe, non-oliguric acute kidney injury and volume overload.

Four months prior to the current presentation, the patient went to the emergency department with left flank pain and bilateral lower extremity edema. Investigations at that time revealed left renal vein thrombosis (diagnosed via Doppler ultrasonography) along with nephrotic range proteinuria (urine albumin-to-creatinine ratio [UACR] 7900 mg/g) and hypoalbuminemia (2.3 g/dL) but with preserved kidney function (serum creatinine 0.76 mg/dL, estimated glomerular filtration rate [eGFR] 100 mL/min/1.73m^2^). She was started on therapeutic anticoagulation, irbesartan, and furosemide and was referred for urgent outpatient nephrology evaluation. At the nephrology visit one week later, urine microscopy revealed no microscopic hematuria or cellular casts. Serologic evaluation included normal C3 and C4; negative antinuclear antibody (ANA), ANCA, anti-GBM, and PLA2R antibody titers; serum and urine protein electrophoresis with no monoclonal bands; and negative human immunodeficiency virus (HIV), hepatitis B, and hepatitis C serologies. The patient denied any non-steroidal anti-inflammatory drug (NSAID) use.

**Fig. 1:**
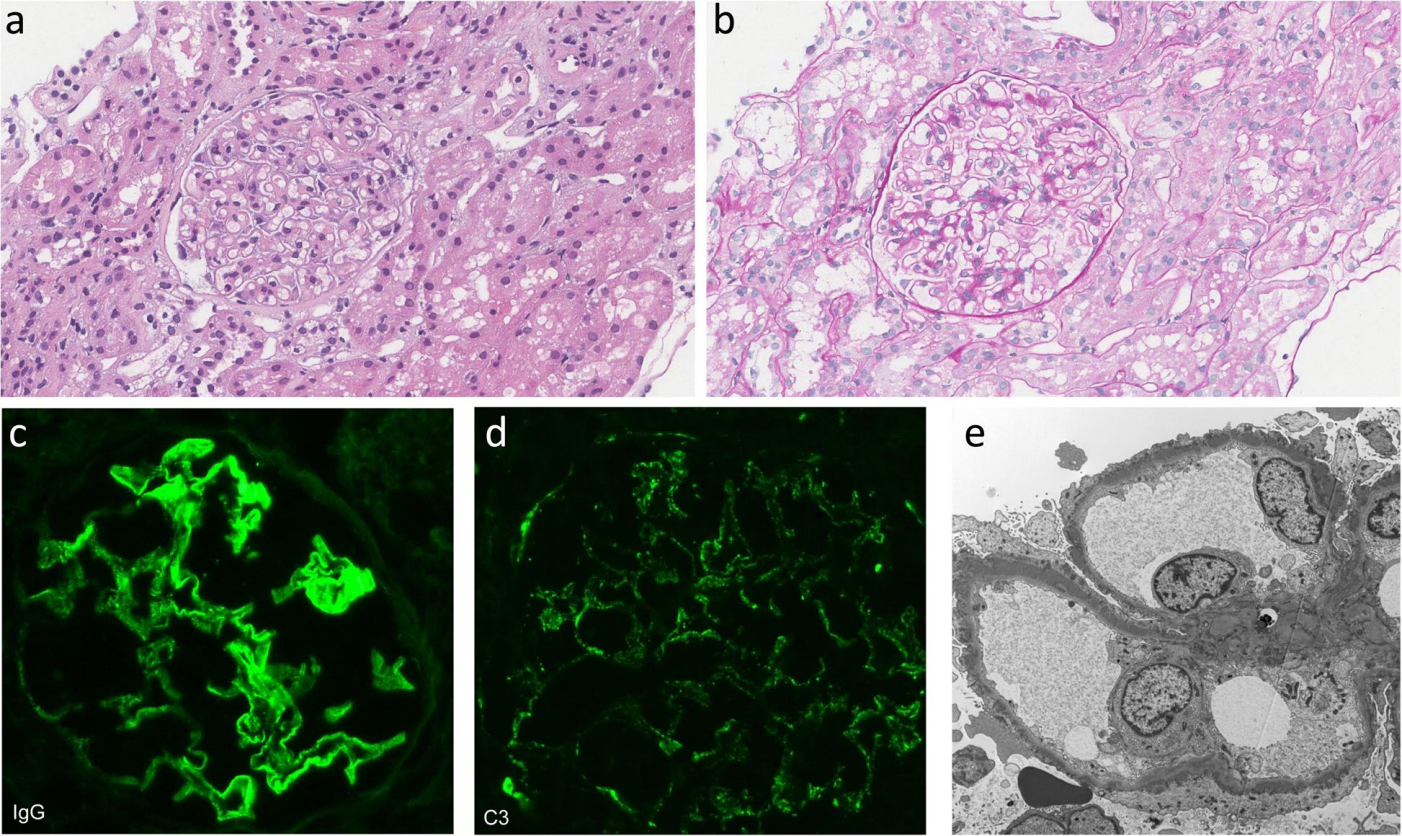
First Kidney Biopsy. **a** Hematoxylin and eosin (H&E) stain shows glomeruli with rigid and mildly thickened capillary walls. **b** PAS reveals glomerulus with rigid and mildly thickened capillary walls. **c**, **d** Immunofluorescence demonstrates 3 + IgG and 2 + C3 granular staining with capillary loop predominance. **e** Electron microscopy shows numerous electron dense subepithelial deposits with diffuse overlying effacement of the podocyte foot processes

The following week, the patient underwent a kidney biopsy (Fig. 1). Specific details regarding the microscopy methods are listed within the Supplementary Appendix. Light microscopy showed 1/21 glomeruli globally sclerosed with the remaining glomeruli having thickened and rigid capillary walls. There was no significant interstitial fibrosis or tubular atrophy, and no crescents were seen. Immunofluorescence demonstrated granular staining in the capillary loops for C3, IgG, kappa, and lambda; IgA and IgM were negative. Electron microscopy revealed numerous subepithelial electron dense deposits with diffuse overlying foot process effacement and no significant mesangial deposits. PLA2R staining on the biopsy was negative. A diagnosis of membranous nephropathy was made. Based on the diagnosis of non-PLA2R-mediated membranous nephropathy, a malignancy work-up was then performed which included computed tomography scans of the chest, abdomen, and pelvis along with mammography and Pap testing all of which were unremarkable. With no secondary causes identified, her case of membranous nephropathy was deemed primary in nature. Despite the associated venous thromboembolism, the decision was made to proceed with a six month observation period (while maintained on irbesartan, furosemide, and warfarin) prior to starting immunosuppression as the patient’s condition in regard to volume overload improved and her eGFR remained normal.

Four months later, the patient returned to the emergency department after two weeks of low-grade fever, cough, dyspnea, and green-tinged sputum. Chest X-ray revealed diffuse airspace disease suggestive of pneumonia. Sputum and blood cultures were collected only after antibiotics and demonstrated no growth. She was treated for community acquired pneumonia with moxifloxacin. Her symptoms gradually improved over the next week. However, over the subsequent two weeks, she developed rapidly worsening dyspnea, hypoxia, and volume overload with a 17 pound weight gain. She was admitted to the hospital where her serum creatinine had increased to 4.01 mg/dL though she remained non-oliguric. She was also increasingly proteinuric (UACR 21,430 mg/g) and hypoalbuminemic (1.9 g/dL). Urine microscopy now revealed dysmorphic red blood cells with a few red blood cell casts. Serologic evaluation now demonstrated a low C3 level (0.32 g/L; reference range 0.90–1.80 g/L) while the C4 level remained normal. ANCA, anti-GBM, and PLA2R antibody titers remained negative. Streptozyme testing was negative. Blood, urine, and sputum cultures were negative. Doppler ultrasonography showed patent bilateral renal veins with resolution of the prior left renal vein thrombosis (notably, this could not definitively rule out residual renal vein thrombosis the given limited sensitivity and specificity of ultrasonography) [8]. A ventilation/perfusion scan was low probability for a pulmonary embolism. Her hypoxia and volume overload were refractory to aggressive intravenous diuresis with concurrent intravenous hypertonic albumin administration, and she was started on hemodialysis. Given her atypical clinical course and suspicion for the development of a rapidly progressive glomerulonephritis, a repeat kidney biopsy was pursued.

**Fig. 2:**
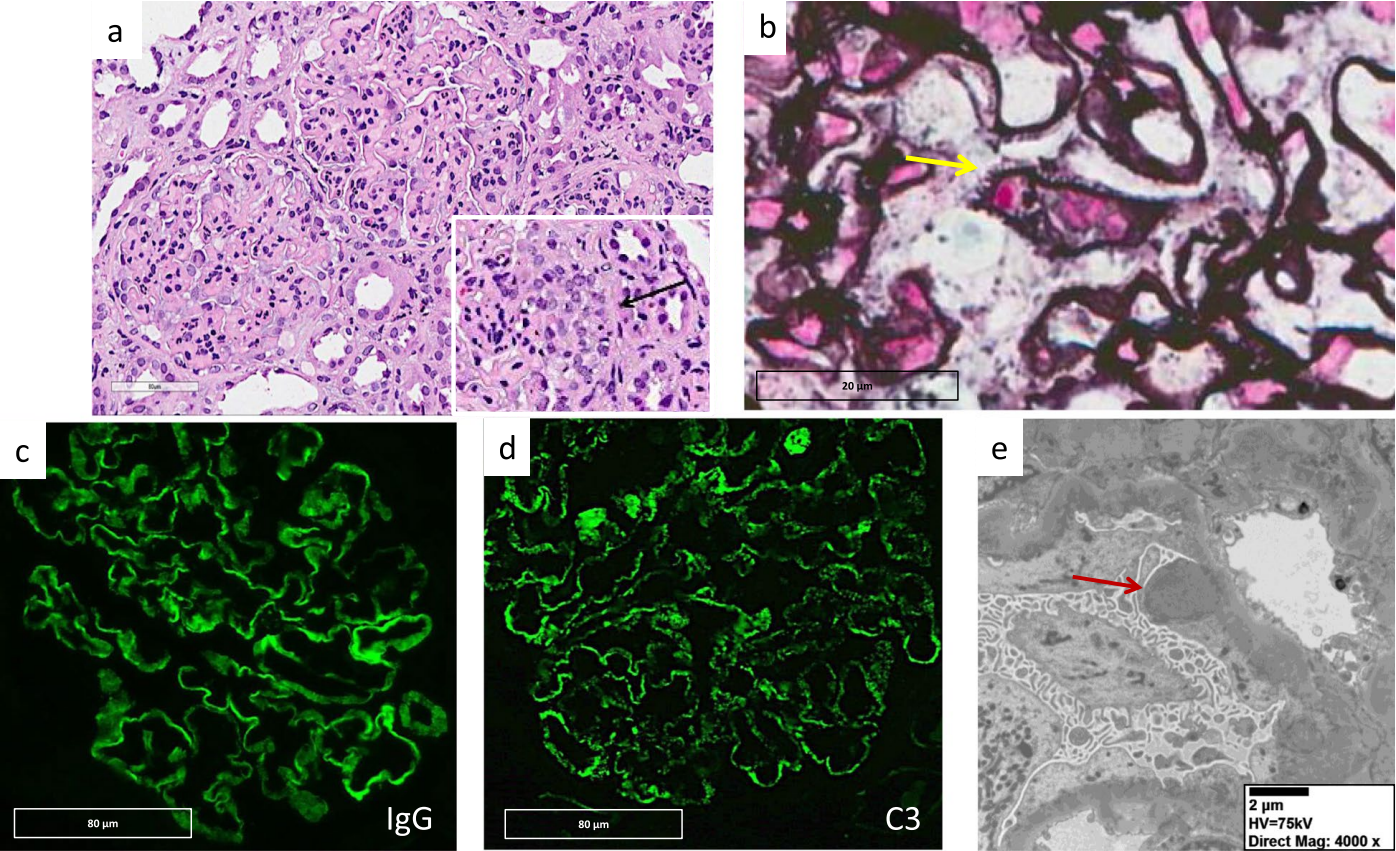
Second Kidney Biopsy. **a** Hematoxylin and eosin (H&E) stain shows glomeruli with endocapillary hypercellularity including neutrophils and eosinophils. A few glomeruli showed cellular crescents (black arrow). **b** Spike lesions were noted on the silver stain (yellow arrow). **c**, **d** Granular and global staining of IgG and C3, predominantly on the capillary loops on immunofluorescence. **e** Electron microscopy shows numerous electron dense subepithelial deposits with diffuse overlying effacement of the podocyte foot processes. Several “hump” lesions were identified (red arrow)

The repeat biopsy (Fig. 2) demonstrated the following set of findings overlaying a background of membranous nephropathy: endocapillary hypercellularity with a neutrophil predominance, mesangial proliferation, endothelial cell swelling, and active cellular crescents in 6/32 glomeruli. Zero of 32 glomeruli were globally sclerosed with no significant tubular atrophy. The immunofluorescence pattern was unchanged with granular staining in the capillary loops for C3, IgG, kappa, and lambda; IgA and IgM were negative. Electron microscopy now showed numerous subepithelial and scattered mesangial electron dense deposits along with several ‘hump’ lesions. Based on these findings, a diagnosis of post-infectious glomerulonephritis (PIGN, presumed to be related to her recent pneumonia) superimposed upon membranous nephropathy was made.

Due to the combination of a lack of improvement of membranous nephropathy during the observation window and the thought that the anti-inflammatory effects of corticosteroids may provide benefit during her acute presentation, treatment was started using the ‘modified Ponticelli’ regimen with corticosteroids and cyclophosphamide (dosed daily at 1.5 mg/kg rather than cyclical). Within two weeks of starting immunosuppression, she was no longer dialysis dependent and her volume status improved while gradually tapering off diuretics. One month post-discharge, her serum creatinine had improved to 1.36 mg/dL, UACR had improved but remained in a nephrotic range at 11,044 mg/g, and serum albumin had increased to 3.1 g/dL. One year post-discharge (and 6 months after completing immunosuppression), her serum creatinine had normalized at 0.72 mg/dL, UACR had declined to a non-nephrotic range at 429 mg/g, and serum albumin had normalized at 4.2 g/dL.

## Discussion and conclusions

This patient’s initial presentation was typical of classic primary membranous nephropathy with nephrotic-range proteinuria, edema, hypoalbuminemia, and venous thromboembolism which corresponded to the classic kidney biopsy findings. However, the sudden and rapid decline in kidney function during a six month observation period, in addition to a newly active urinary sediment, was highly atypical and raised concern for transformation into a crescentic form of membranous nephropathy. There are a growing number of reports of crescentic membranous nephropathy which are typically associated with co-existence of ANCA and anti-GBM disease (repeat serologies for which were negative in this case) and have a variable prognosis with approximately 40% of such patients progressing to end-stage kidney disease [4–6]. However, this case highlights the value in pursuing a repeat kidney biopsy when the course of nephrotic syndrome is atypical as it provided an alternate explanation for this patient’s sudden decline – the development of PIGN superimposed upon a background of pre-existing untreated membranous nephropathy. To our knowledge, this is the first report of the simultaneous occurrence of these two glomerular disease processes.

Among otherwise healthy young adults with PIGN, the need for dialysis is uncommon and the likelihood of complete recovery is high [9]. This may reflect the influence of kidney functional reserve – the ability of the kidneys to hemodynamically adapt during stress to maintain glomerular filtration rate (GFR) [10]. When this physiologic ‘reserve’ becomes depleted, the ability of the kidneys to compensate to such a stressor may be diminished. For instance, the course of PIGN is more severe with a lower likelihood of complete recovery in older populations where nephron mass is reduced [11] and among patients with additional concurrent glomerular or tubulointerstital disease [12–14]. Perhaps the pre-existing kidney damage in these patients heightens the risk for severe acute kidney injury due to an inability to hemodynamically regulate GFR with the added disease stressor of PIGN. In the present case, the co-existing glomerular damage from active and untreated membranous glomerulopathy may have restricted this young patient’s ability to maintain adequate GFR with the acute stressor of PIGN and the combination of the two glomerular processes led to severe acute kidney injury requiring dialysis.

This case also highlights the debate around which patients with membranous nephropathy should receive immunosuppressive therapy at initial presentation versus which patients should be observed. The pros (e.g., expedited kidney improvement, lower venous thromboembolism risk, etc.) must be weighed against the cons (e.g., heightened infection risk, medication side effects, etc.) on a case-by-case basis. In this instance, despite a normal eGFR and appearing ‘clinically well’ at presentation, the patient had severe nephrotic range proteinuria and had already suffered a major nephrotic syndrome-associated complication of venous thromboembolism which many nephrologists would likely consider as justification for immunosuppression [2]. Perhaps earlier initiation of immunosuppression would have replenished her kidney functional reserve such that she would have been less susceptible to the added stressor of PIGN and limited the severity of her clinical course. Alternatively, earlier immunosuppression may have potentiated the severity of her pneumonia course.

In conclusion, we report the case of a young patient with a classic initial presentation of membranous nephropathy with a sudden and rapid deterioration in kidney function requiring hemodialysis during a six month observation period. The sudden worsening was related to superimposed PIGN likely related to a recent community acquired pneumonia with perhaps reduced kidney functional reserve due to untreated membranous nephropathy. Concurrent glomerular diseases, such as PIGN, should be considered in the differential diagnosis for rapidly progressive membranous nephropathy, and repeat kidney biopsy may be necessary to provide an accurate diagnosis and direct patient care.

## Supplementary Information

**Additional file 1.**

## Data Availability

Data sharing is not applicable.

## Abbreviations

ANA: Antinuclear antibody
ANCA: Antineutrophil cytoplasmic antibody
eGFR: Estimated glomerular filtration rate
GBM: Glomerular basement membrane
GFR: Glomerular filtration rate
HIV: Human immunodeficiency virus
NSAID: Non-steroidal anti-inflammatory drug
PIGN: Post-infectious glomerulonephritis
PLA2R: Phospholipase A2 receptor
THSD7A: Thrombospondin type-1 domain-containing 7A
UACR: Urine albumin-to-creatinine ratio
