# Supplementary material for: The value of repeat kidney biopsy during an atypical course of membranous nephropathy
Source: BMC Nephrol. 2022 Jul 7;23:240. doi: 10.1186/s12882-022-02863-y (PMC9260970; doi:10.1186/s12882-022-02863-y)
Supplement: Supplementary file 1 — Additional file 1. [file 12882_2022_2863_MOESM1_ESM.docx]

**SUPPLEMENTARY APPENDIX:**

**The Value OF Repeat Kidney Biopsy During an Atypical Course of Membranous Nephropathy**

**Correspondence To:**

Dr. Gregory L. Hundemer

Ottawa Hospital – Riverside Campus

1967 Riverside Drive, Office 5-33

Ottawa, Ontario

Canada

K1H 7W9

Phone: (613) 738-8400

Fax: (613) 738-8337

E-mail: [ghundemer@toh.ca](mailto:ghundemer@toh.ca)

**MICROSCOPY DETAILS**

**Light Microscopy** - The glass slides were digitized using the Aperio scanner and the images taken using the Aperio ImageScope software. No enhancement of the images was performed. All images are at 40X magnification.

**Immunofluorescence** - All images are at 40X magnification.

**Electron Microscopy** – All electron microscopy images are original and captured using an AMT XR-41 camera. The scale and resolution are included on the images in the manuscript.
